# Effects of Assisting Solvents on Purification Efficiency in the Layer Melt Crystallization of Glycerol

**DOI:** 10.1021/acs.cgd.3c01207

**Published:** 2024-02-21

**Authors:** Mitra Ila, Kim Miikki, Marjatta Louhi-Kultanen

**Affiliations:** † Department of Chemical and Metallurgical Engineering, School of Chemical Engineering, Aalto University, Espoo 02150, Finland; ‡ School of Chemical Engineering, Aalto University, Espoo 02150, Finland

## Abstract

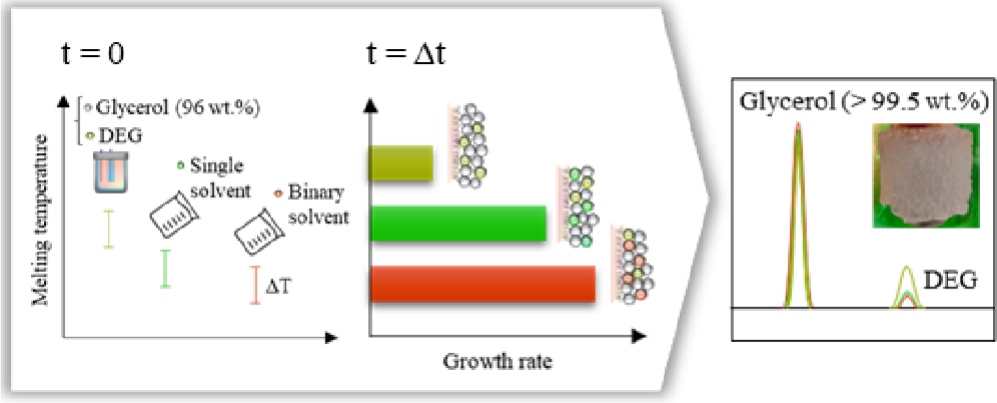

The efficiency of layer melt crystallization for the selective separation of glycerol from a glycerol–diethylene glycol mixture containing 4 wt % of diethylene glycol was evaluated using solvent-free and solvent-aided approaches. The effect of 1-butanol and the binary solvent of 1-butanol and acetone with a total molar composition of 25 mol % on the crystal growth kinetics and the purity of the final product was studied at different undercooling degrees and crystallization yields. The melting point temperature of the mixtures was predicted by using the modified UNIFAC Dortmund model. The addition of both a single and binary solvents significantly increased the crystal growth rate and purity of the final product compared to crystallization from the solvent-free mixture. Additionally, higher crystal growth rates and product purity were observed in the binary solvent system compared to those in the single solvent system at the same degree of undercooling. The second stage of solvent-aided crystallization resulted in glycerol with a purity above 99.5 wt %, depending on the type of solvent used and the rate of crystal growth.

## Abstract 2

Crystal purity higher than 99.5 wt % obtained by two-step melt crystallization. Layer melt crystallization of glycerol requires seeding. Binary solvent of 1-butanol and acetone increased crystal purity. Image analysis successfully used to monitor inline crystal growth.

## 1 Introduction

Crude glycerol is a byproduct of several biorefineries, such as biodiesel production^1^ and conversion of nonedible biomass into renewable chemicals. Efficient use of glycerol promotes sustainable development and reduces waste disposal. Glycerol can be used as a renewable resource for diverse applications such as pharmaceuticals, cosmetics, and food products, or as a raw material for the production of other chemicals^1,2^ depending on its grade. Purification of glycerol by distillation requires vacuum operation due to its high boiling point and sensitivity to high temperatures.^3,4^ Recent efforts have been focused on optimizing the vacuum distillation process using computational methods to reduce the cost associated with this purification step.^4^ Nevertheless, the overall efficiency of purification methods relies on the impurity content of crude glycerol.

Diethylene glycol (DEG) is one of the major impurities coproduced with glycerol during the conversion of plant-based sugars into renewable chemicals. The presence of impurities in glycerol, which is used as a component in pharmaceuticals and food products, poses a potential health risk. The European Pharmacopoeia specifies a limit of maximum 0.1% for the amount of DEG in glycerol as an impurity.^5^ In the context of purification of glycerol from such organic impurities, the distinct melting points of individual compounds offer advantages for purification as a basis for the melt crystallization method. The aim of this work is to evaluate melt crystallization, a highly selective and energy efficient separation technique, to produce high purity glycerol for use in various industrial applications.

Eisenbart et al.^6^ studied the effect of adding 5–30 wt % of 1-butanol on the purification efficiency of layer crystallization for the production of highly dry glycerol. It was shown that the addition of solvent significantly increased the purity of the layer crystallization product compared to crystallization without the use of solvent. A higher solvent concentration led to a higher purity of the final product. Earlier research by Hass and Patterson^7^ showed that 1-butanol was one of the most suitable solvents among the 30 solvents screened to increase the growth rate of glycerol crystals. Nevertheless, most of the examined solvents with complete or partial solubility in glycerol were polar protic solvents.

Acetone, a polar aprotic solvent, has a viscosity lower than 1-butanol. The viscosity of acetone and 1-butanol at 298.15 K is 0.308 mPa s and 2.599 mPa s, respectively.^8^ Furthermore, the high volatility characteristic of acetone facilitates the solvent recovery process. However, acetone is sparingly soluble in glycerol. Therefore, in this work, a mixture of 1-butanol and acetone was introduced as a new binary solvent for solvent-aided layer melt crystallization. A comparative analysis was carried out to assess the efficiency of static layer melt crystallization for the purification of glycerol from DEG with and without using 1-butanol and the new binary solvent as assisting solvents.

## 2 Materials and Methods

### 2.1 Chemicals

Glycerol (>99%), diethylene glycol (99%), 1-butanol (>99%), and acetone (99.5%) were purchased from Thermo Fisher Scientific, Inc. and manufactured by Thermo Scientific Chemicals. All the chemicals were used without further purification.

### 2.2 Seeding Preparations

Gibson and Giauque^9^ and Eisenbart and Ulrich^10^ showed that glycerol crystallization without seeding occurs by cooling glycerol with liquid air or liquid nitrogen followed by slow warming of the cooled sample. For a gradual increase in temperature, Eisenbart and Ulrich^10^ stored samples at 248 K inside a styrofoam box. Using the same method, glycerol was rapidly cooled and solidified using liquid nitrogen. The solidified samples were stored in a styrofoam box in a refrigerator at a temperature of 255.15 K. The gradual increase in the temperature of the sample led to the formation of the glycerol crystals, which were used for seeding.

### 2.3 Cold Finger Set Up for Layer Melt Crystallization Studies

**Figure 1:**
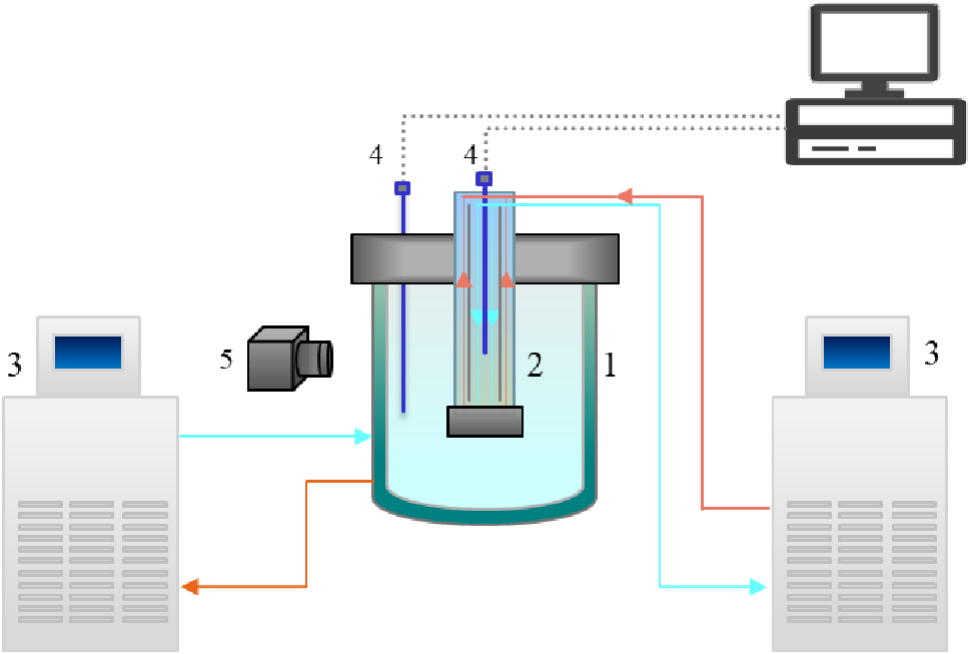
Schematic overview of static layer melt crystallization setup: (1) double jacket, (2) cold finger, (3) thermostat, (4) thermocouple, and (5) camera.

The static layer crystallization set up consists of a 100 mL jacketed vessel and a cold finger with a diameter of 9.6 mm and a length of 18 mm connected to thermostats (Figure 1). The temperature sensors located inside the melt and cold finger were used to adjust the operating temperatures. A thin crystalline layer of pure glycerol was formed on the cold finger to induce crystallization upon contact with the melt. The crystal layer was formed by immersing the cold finger into glycerol at subzero temperatures. The cold finger was removed, and a layer of viscous glycerol remained on the surface. The layer was rapidly crystallized by scratching the surface with a seed crystal. The measurement of the crystal growth rate perpendicular to this crystal layer was then started using image analysis.

The efficiency of the layer melt crystallization for the purification of glycerol was examined for a mixture containing 4 wt % DEG. Solvent-aided melt crystallization was carried out by adding a total solvent concentration of 25 mol % in both single and binary solvent systems to obtain the appropriate level of viscosity reduction and maintaining adequate solubility in the original melt. Due to the low solubility of acetone in glycerol, a mixture of acetone and 1-butanol was used as the binary solvent. The amount of acetone and 1-butanol used was 15 and 10 mol % of the total feed with a higher concentration of acetone compared with 1-butanol to emphasize acetone influential factors on the melt crystallization efficiency.

### 2.4 Image Capturing and Image Processing

The modular camera system comprising a Raspberry Pi HQ Camera Module with 12.3 megapixels still resolution and a Raspberry Pi 4/4 GB platform was used to capture images of the growing crystal layer. Detailed information about the software is given by Miikki et al.^11^

In general, auto white balance (AWB) values should be locked if the lighting conditions are invariable and detection of color changes is desired. Statistical analyses of such methods are given in ref (12). However, when detection of changes in shapes and dimensions is required, the AWB calibration can be omitted, and the use of the camera AWB algorithm is acceptable. Hence, the AWB camera was used in this work for time-lapse photography.

The crystal length measurement method consists of image processing, shape detection, and calibration based on the pixel to real distance conversion factor (pixels/mm) using the OpenCV library in Python.

### 2.5 Analysis Methods

#### 2.5.1 HPLC

Product purity was measured with a Waters 2695 high performance liquid chromatograph (HPLC) equipped with a refractive index detector and a Shodex Sugar SC1011 column (8.0 mm inner diameter and 300 mm length). The oven temperature was set at 70 °C, and water was used as the eluent. The injection volume and flow rate were set to 10 μL and 1 mL/min, respectively (see Supporting Information).

#### 2.5.2 Viscosity Measurement

The viscosity of the mixtures was measured with an Anton Paar Physica MCR 301 rheometer. An ultrasonic bath was used to remove air bubbles from the mixture prior to viscosity measurement. A slow shear rate of 10 s^–1^ in the concentric cylinder geometry was used to avoid the formation of air bubbles during measurement.

#### 2.5.3 Optical Microscopy

The crystal growth of glycerol single crystals in the melt was monitored by an optical microscope (Olympus BX53M) equipped with a temperature control system.

## 3 Results and Discussion

### 3.1 Effect of Impurity and Assisting Solvents on Melting Point Depression and Viscosity

The modified UNIFAC-Dortmund (UNIFAC-DMD) group contribution model^13^ was used to predict the melting point depression of liquid phases based on the interactions between the functional groups of the system components. Given the complexities of measuring the solid–liquid equilibrium for glycerol mixtures, the melting point temperature estimated by this model was used as a reference point for the applied undercooling degree.

The functional groups of glycerol include two primary (−OH) and one secondary (−OH) hydroxyl groups, two CH_2_ groups, and one CH group. DEG is defined by one CH_2_O, two primary (−OH), and three CH_2_ groups. Acetone is assigned with two functional groups, including CH_3_CO and CH_3_ groups. 1-butanol has one primary (−OH) hydroxyl group, three CH_2_, and a CH_3_ group. The melting point of the mixtures is calculated in relation to the activity coefficient, γ_*i*_, and the composition of component *i* in the liquid phase using eq 1, assuming the formation of a pure solid phase.^14^ The enthalpy of fusion, Δ*H*_m_, and melting point of pure glycerol, *T*_m_, are 18.280 kJ/mol and 291.33 K, respectively.^15^
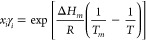
1

Table 1 shows the predicted melting point depression in the solvent-free and solvent-aided mixtures and the viscosity measured at their melting point. The predicted results show a difference of about 1.2 K between the melting temperatures of the systems with single and binary solvents, while the binary solvent system exhibits a lower viscosity at a total equivalent composition of 25 mol %.

**Table 1:** Melting Temperature Predicted by UNIFAC-DMD Model and Measured Viscosity for Systems Consisting of Glycerol [1], DEG [2], 1-Butanol [3], and Acetone [4] Quantified by Mole Fraction

	*x*_1_ (−)	*x*_2_ (−)	*x*_3_ (−)	*x*_4_ (−)	melting point temperature (UNIFAC-DMD) (K)	viscosity (mPa·s)
solvent-free	0.9651	0.0349	0	0	289.98	1443
single solvent	0.7238	0.0262	0.25	0	283.37	553
binary solvent	0.7238	0.0262	0.1	0.15	282.18	394

### 3.2 Purification Efficiency in Solvent-Free and Solvent-Aided Layer Melt Crystallization

The purification efficiency of static layer melt crystallization was evaluated in the cold finger setup. The temperature of the cold finger was set at 5, 10, and 15 K below the melting point of the mixture and kept constant for 2 h before gradually cooling it down to maintain the crystal growth rate during crystallization. The temperature of the bulk melt was adjusted to 1–1.5 K higher than the melting point of the feed. The crystal growth rate at different crystallization times was measured by using the image analysis technique. Due to the nonuniform growth of the crystal layer in the solvent-free system (see Section 3.5), the average growth rate of this system was determined for the beginning of the crystallization. The purity of the product on a solvent-free basis was measured for the crystalline layer with a thickness of 2.5–3.5 mm. The effective distribution coefficient of the impurity was defined as the ratio between the impurity content in the crystal layer and the impurity content present in the feed. A second crystallization stage was carried out using a mixture with the same impurity content as the product obtained from the first stage at the same undercooling degree. Crystallization from solvent-aided systems was followed by the formation of two liquid phases due to the limited solubility of both the single and binary solvents in the glycerol-DEG mixture. The purity of the crystal layer at higher crystallization yields during liquid–liquid phase transition is addressed in Section 3.3.

**Figure 2:**
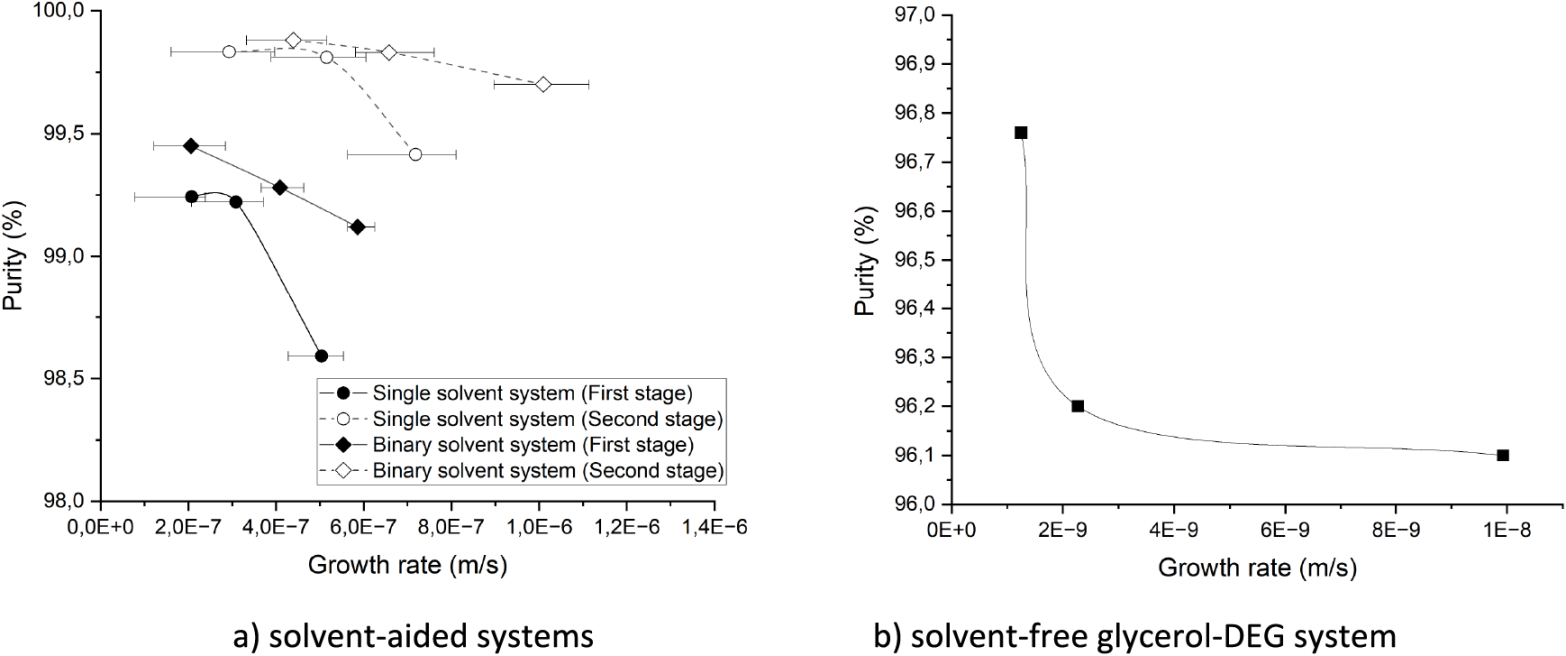
Crystal growth rate versus crystal purity on a solvent-free basis. The data marked by squares, circles, and diamonds represent solvent-free, single solvent, and binary solvent systems, respectively. The straight and dashed lines show the growth rates in the first and second stages of crystallization, respectively.

**Figure 3:**
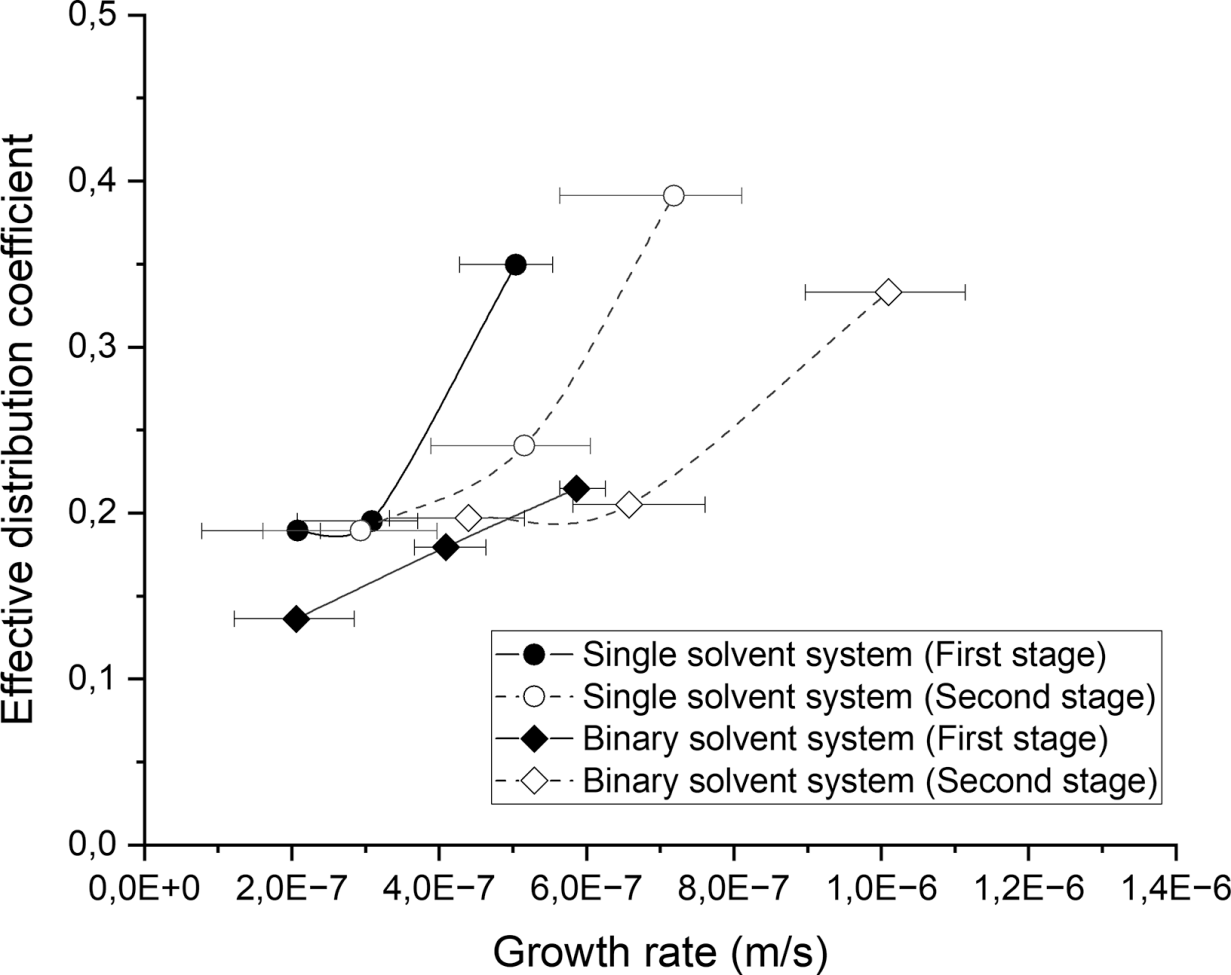
Effective distribution coefficient of the product vs crystal growth rate. The data marked by circles and diamonds represent single solvent and binary solvent systems, respectively. The straight line and dashed line show the growth rates in the first and second stages of crystallization, respectively.

Figure 2 a,b shows that crystal layer purity of 99.1, 98.6, and 96.1 wt.% on a solvent-free basis was obtained at the highest crystal growth rate in each binary solvent, single solvent, and solvent-free system, respectively, after the first crystallization stage. Although a slow crystal growth rate favors the purification efficiency of crystallization, higher product purity was achieved in the solvent-aided mixtures with higher crystal growth rates compared with the glycerol-DEG system. Similarly, higher purity levels were obtained in crystallization from the binary solvent system than from the single solvent system despite higher crystal growth rates. This may indicate a better depletion efficiency of the rejected impurity during crystal growth. Depletion of impurities involves their desorption from the crystal interface followed by their transport from the interface to the bulk melt.^16^ Therefore, the lower viscosity of the mother liquor in the binary solvent system is one of the key factors affecting the purification efficiency in static layer melt crystallization. The same scenario applies to the purification efficiency of solvent-aided systems in the second stage of crystallization. Although the crystal growth rate increased in the second crystallization stage, a minor increase of up to 0.06 and 0.12 was observed in the impurity distribution coefficient of the single solvent and binary solvent systems, respectively (Figure 3).

Nevertheless, the negative impact of higher crystal growth rates on the crystal purity is evident in each individual system. However, the product obtained from the single solvent system showed a larger decline in purity, as the crystal growth rate increased in the first crystallization stage compared to that of the binary solvent system. This further shows that the depletion and transport of impurities rejected on the crystal surface in the single solvent system are not fast enough compared to the crystal growth rate, which leads to an increase in the impurity concentration on the crystal surface, resulting in a higher chance of impurity incorporation. The effect of crystal growth rate and mass transfer on crystal purity in static layer melt crystallization has been discussed in different studies.^17,18^ Guardani et al.^17^ explained the higher effective distribution coefficient of the crystal layer in static mode crystallization due to the increase in impurity concentration at the solid–liquid interface, as a result of the slow transport of impurities from the interface to the bulk melt.

### 3.3 Influence of Crystallization Yield on Purity in Solvent-Aided Systems

Crystallization from both solvent-aided systems was followed by the formation of two liquid phases. In the cold finger setup, a thin layer of the second liquid phase appeared above the main body of the bulk melt (see Supporting Information) where both crystallization and liquid–liquid phase transitions occurred simultaneously. To evaluate the effect of the formation of two liquid phases on the purification efficiency, the purity of the crystal layer was measured as a function of crystallization yield when different amounts of glycerol were crystallized over time. The crystallization yield is defined as the ratio between the amount of crystallized glycerol and the initial amount of glycerol in the feed. For this purpose, layer crystallization was carried out inside a beaker with a 38 mm diameter immersed in a cooling bath with the crystal layer growing on the bottom of the beaker. A thin layer of pure glycerol was crystallized at the bottom of the beaker to initiate crystallization. Using the equation below, the crystal growth rate was determined at an initial undercooling of 15 K:
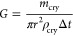
2where a glycerol solid density value of 1335.5 kg/m^3^ at 291.15 K^15^ was assumed for the density of the crystal, ρ_cry_·*m*_cry_ is the mass of the crystal growing at the bottom of the beaker, with radius *r* at crystallization time Δ*t*.

**Figure 4:**
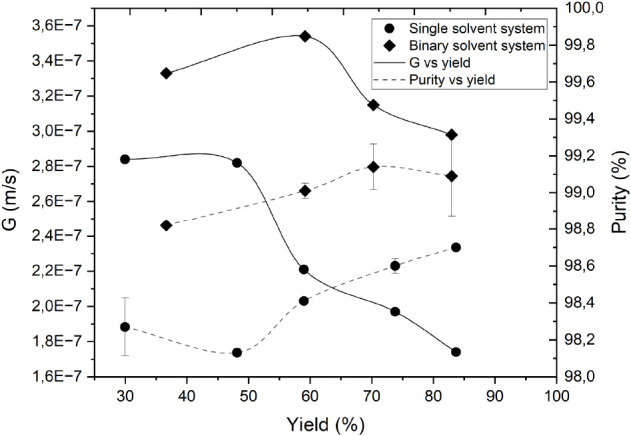
Purity of the product on a solvent-free basis at different crystallization yields obtained at an initial undercooling degree of 15 K. The solid line shows the average crystal growth rate versus yield, and the dashed line shows the product purity versus yield. The data marked by circles and diamonds represent single solvent and binary solvent systems, respectively.

Figure 4 shows that the crystal growth rate in both the single and binary solvent systems was lower than in the cold finger setup. The main differences between these two setups are the surface area for the crystal growth and the adjusted temperature of the bulk melt. Immersing the beaker in the cooling bath lowers the initial temperature of the melt to the adjusted undercooling degree, while in the cold finger setup the temperature is set above the melting point of the feed. Changes in temperature-dependent physical properties of the melt, such as the diffusion coefficient and viscosity, can affect the crystal growth rate. A higher melt viscosity also accounts for the higher incorporation of impurities. Despite these differences, Figure 4 shows that the purity level in the binary solvent system increased with a minor change in the crystal growth rate in crystallization yields up to 80%. This implies that the liquid–liquid phase transition during crystallization had no notable adverse impact on the purity of the product. A similar conclusion can be drawn for the single solvent system. However, a greater decrease in the crystal growth rate was observed in the single solvent system during progressing crystallization. This may indicate that the influence of increasing impurity concentration in the liquid phase during crystallization is more significant in the single solvent system than in the binary solvent system.

**Figure 5:**
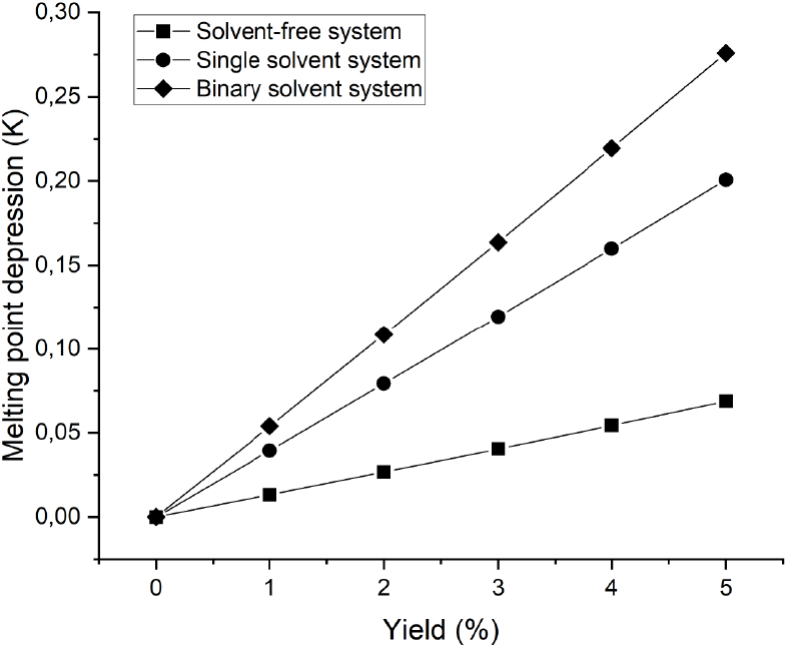
Prediction of melting point depression in the mother liquor during progressing crystallization using the modified UNIFAC Dortmund model. The data marked by squares, circles, and diamonds represent solvent-free, single solvent, and binary solvent systems, respectively.

Nevertheless, solvent-aided layer melt crystallization of glycerol showed efficient purification by maintaining purity while maximizing the process yield.

### 3.4 Effect of Impurity and Solvent(s) on Crystal Growth Rate

The concept of the addition of solvent in crystallization from solution, antisolvent crystallization, is mainly known from the thermodynamic point of view of changing the degree of supersaturation. However, Dighe et al.^19^ investigated the underlying mechanism of antisolvent crystallization by referring to the fact that nucleation and crystal growth rates in different solvents can be different despite the same level of supersaturation. Likewise, the addition of solvent can affect the crystal growth rate in the crystallization from the melt in different ways.

To evaluate the effect of the solvent on the driving force for crystallization, the melting depression of the mother liquor during the progressing crystallization was predicted by the UNIFAC-DMD model, assuming the formation of a pure crystal layer. Solid–liquid equilibrium data were evaluated for different mother liquor compositions at crystallization yields of less than 5% due to the potential formation of two liquid phases in solvent-aided systems at higher yields. During progressing crystallization, the concentration of the impurity and solvent in the mother liquor increases, which lowers the melting point of the bulk melt. Figure 5 shows that a higher degree of subcooling is required in solvent-aided systems to achieve the same equilibrium yield as in the solvent-free system due to the higher depression in the melting point of solvent-aided systems. This also implies that the same degree of undercooling results in a lower driving force (supersaturation) for crystallization in solvent-aided systems. A lower crystal growth rate is expected at a lower driving force unless other underlying factors such as mass transfer, interfacial phenomena, and surface kinetics have an effect.

**Figure 6:**
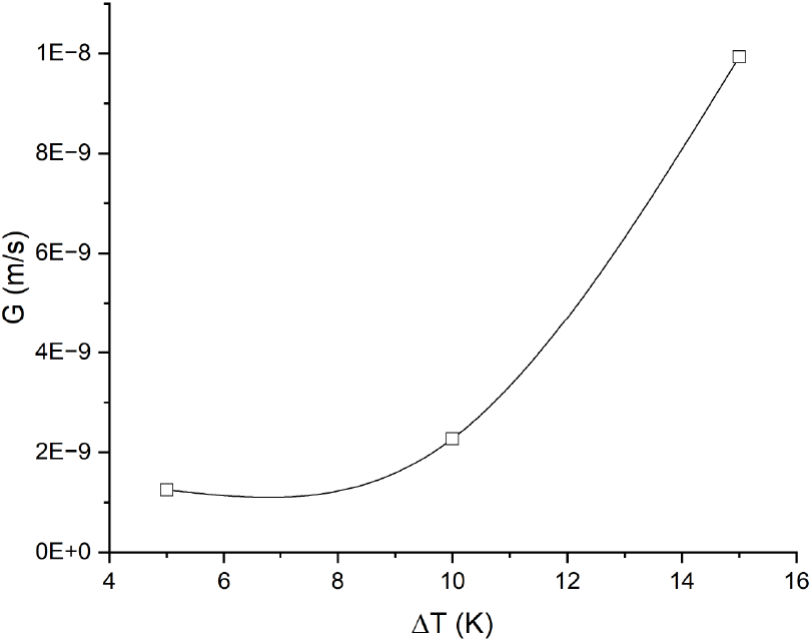
Crystal growth rate in the glycerol-DEG system at different initial undercooling degrees (Δ*T*). The melting point temperature predicted by the modified UNIFAC Dortmund model was the reference point for the undercooling degree.

**Figure 7:**
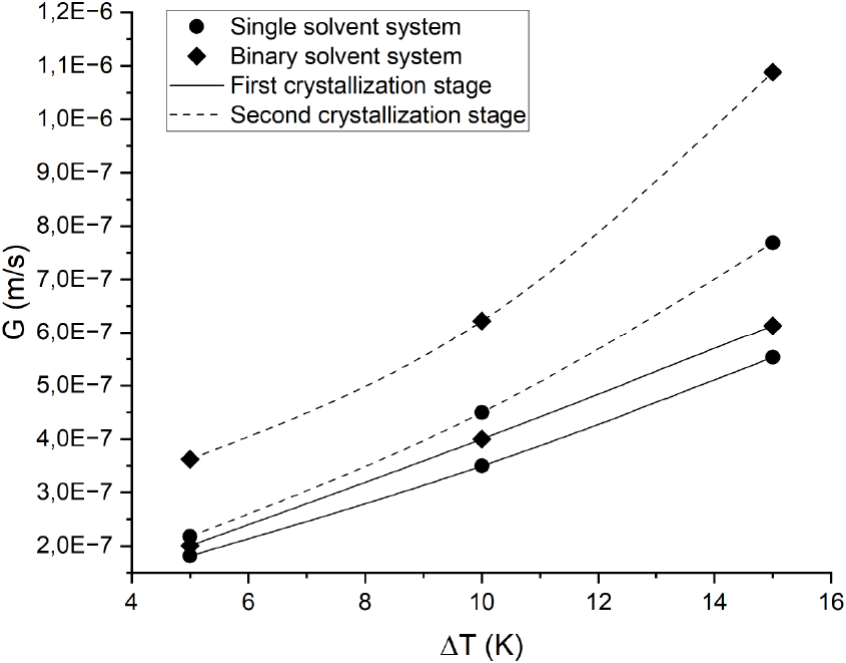
Crystal growth rates in the first and second crystallization stages from solvent-aided systems. The straight line and dashed line show the first and second crystallization stages, respectively. The data marked with circles and diamonds represent single and binary solvent systems, respectively. The total composition of solvents in each system is 25 mol.%. The melting point temperature predicted by the modified UNIFAC Dortmund model was the reference point for the undercooling degrees applied (Δ*T*).

To evaluate the effect of the solvent on the crystal growth kinetics, the crystal growth rate was measured at different undercooling degrees for each system. The average crystal growth rate was measured up to 120 min of crystal growth. Figures 6 and 7 show that the crystal growth rate is significantly higher in both solvent-aided systems than in the solvent-free system, even though a lower crystal growth rate was expected, as the driving force for crystallization was lower at the same degree of undercooling. This suggests that the crystal growth rate is influenced by other factors such as mass transfer, surface integration, or a combination of both.

**Figure 8:**
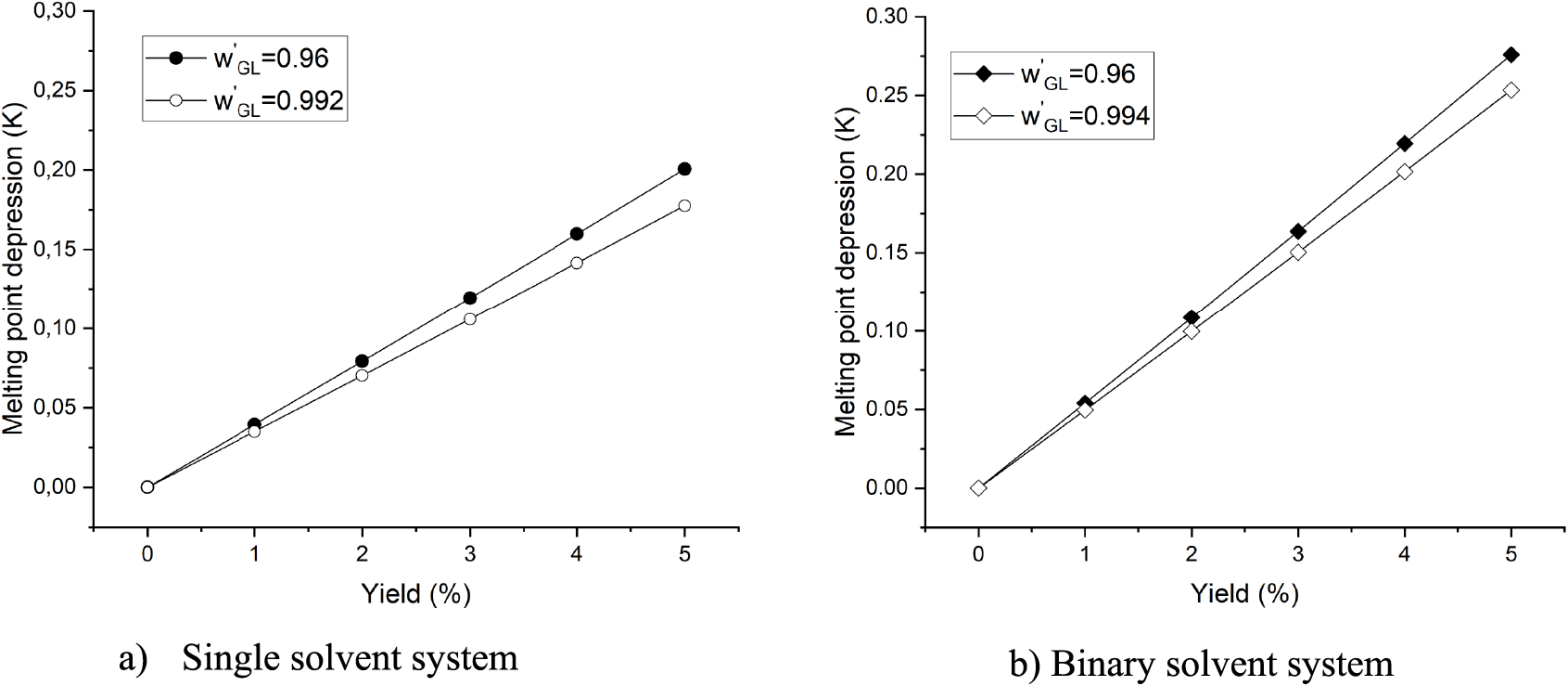
Prediction of the melting point depression of the mother liquor in solvent-aided systems with different impurity concentrations using the modified UNIFAC Dortmund model. The concentration of glycerol in the feed of the second stage of crystallization with the lowest amount of impurity is 99.2 and 99.4 wt % (solvent-free basis, *w′*_GL_) in the single and binary solvent systems, respectively.

Furthermore, Figure 7 shows an increasing trend in the crystal growth rate in the second crystallization stage, where the feed contained a lower impurity concentration than in the first stage. Figure 8 shows a minor difference of up to 0.02 K between the melting point depressions of mixtures with different impurity concentrations. In terms of the driving force for crystallization, it is improbable for this minor difference to be the primary cause of the significant change in the growth rate, as shown in Figure 7. In the context of growth theories, impurities can affect the growth kinetics by physically blocking and reducing the number of kink sites available for the integration of growth units, interfering with the movement of steps across the crystal surface or by changing the interface properties such as interfacial energy.^20^

**Figure 9:**
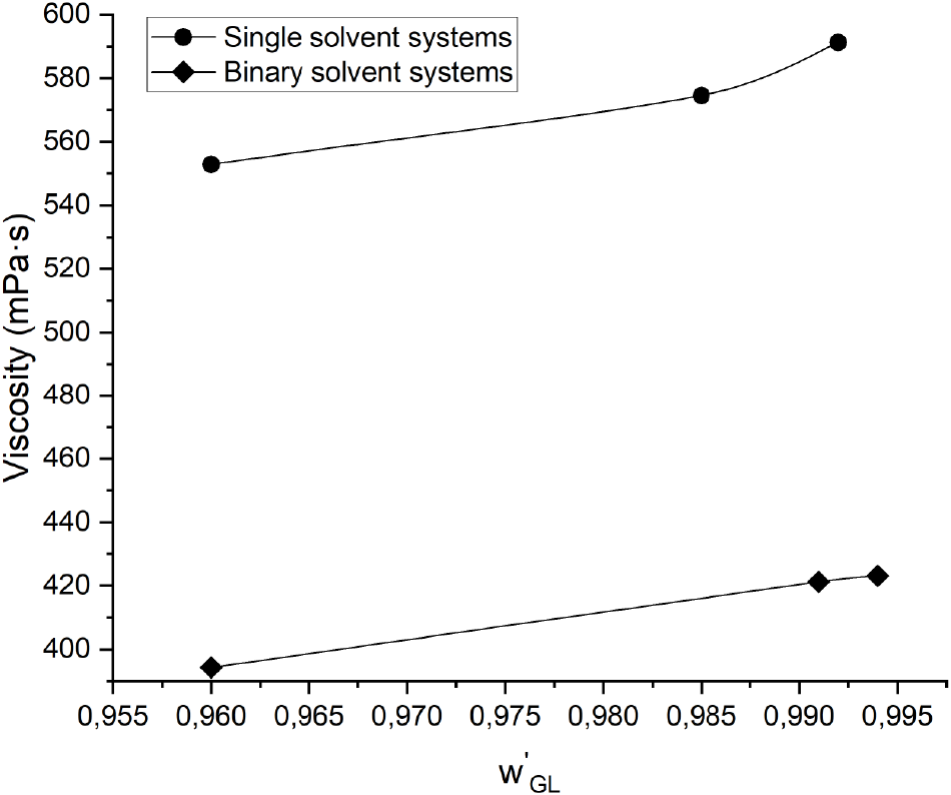
Measured viscosity of the single and binary solvent systems with different impurity contents at the melting point temperature predicted by the modified UNIFAC Dortmund model. The *x*-axis represents the composition of glycerol on a solvent-free basis, *w′*_GL_, with the total solvent composition of 25 mol % in each system. The data marked with circles and diamonds represent single solvent and binary solvent systems, respectively.

Along with the effect of interfacial phenomena, the growth rate can also be influenced by transport phenomena in the melt. Furthermore, high viscosity can be a barrier to the diffusion of building blocks during crystallization.^16^Figure 9 shows that although the viscosity of melts with higher concentrations of DEG was measured at lower temperatures due to the effect of impurity on melting point depression, the viscosity value was lower as DEG has a significantly lower viscosity than glycerol. As a higher impurity concentration decreased viscosity while reducing the overall growth rate, this may imply that the presence of DEG on the crystal surface plays a crucial role in determining the crystal growth rate. Furthermore, the crystal growth rate of glycerol in pure melt and the binary mixture of glycerol and diethylene glycol was measured under the microscope at undercooling of 15 degrees. While different crystal surfaces exhibit different growth rates, the crystal growth rate in the pure melt was 2 to 6 times higher than that in the binary mixture.

Therefore, accumulation of the impurity at the surface of the crystal due to slow transport of impurity from the surface of the crystal to the bulk melt can amplify the negative effect of impurity on the crystal growth rate. Hence, the lower viscosity of the binary solvent system is more favorable for crystal growth than those of the single solvent and solvent-free systems.

### 3.5 Effect of Solvent on Uniform Growth of Crystal Layer

**Figure 10:**
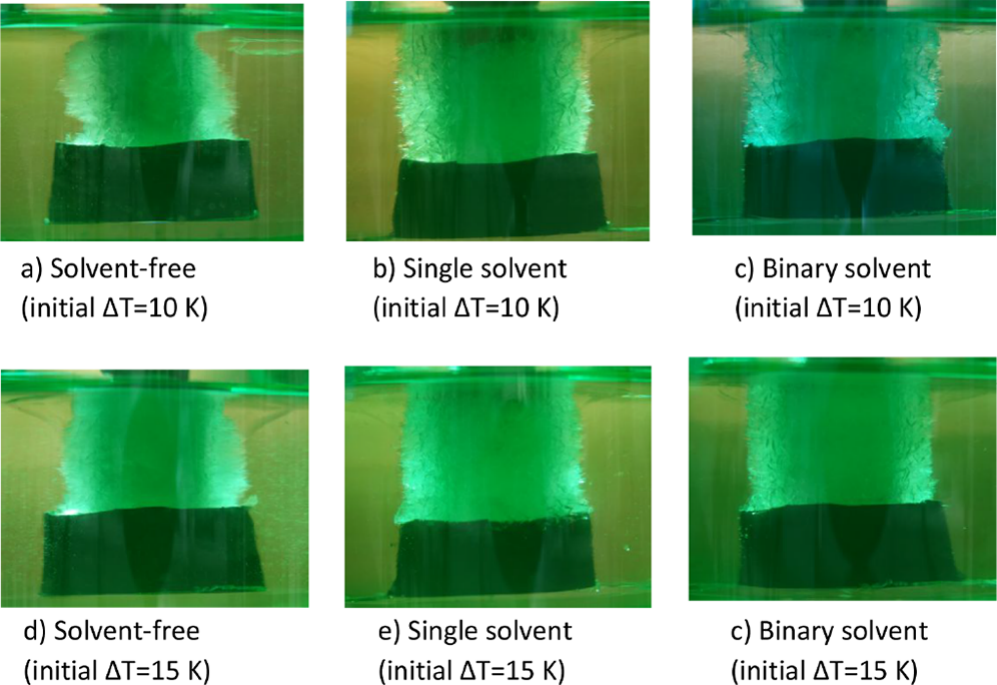
Effect of solvent on the uniform growth of the crystal layer at different undercooling degrees.

Figure 10 shows that the crystal layer obtained from solvent-aided systems grew more evenly compared to the solvent-free melt at different undercooling degrees. Nonuniform growth of the crystal layer can pose challenges in effective control over purity level due to the variation in growth kinetics across the crystal layer, greater probability of inclusion formation, as well as difficulties in separation of residual melt from the crystal layer.

## 4 Conclusion

The effect of impurity and assisting solvent(s) on the separation efficiency of melt crystallization in the purification of glycerol was investigated. Melting point depression during progressing crystallization was predicted in each system using the modified UNIFAC Dortmund model. Monitoring the crystal growth in each individual system at different undercooling degrees showed that the crystal growth rate without addition of any solvent was significantly lower than that with solvent-aided crystallization, while the lowest product purity was obtained in the solvent-free system. Although higher melting point depressions were predicted for the mixtures with binary solvent during progressing crystallization, both crystal growth rate and product purity were higher compared to single solvent and solvent-free systems at the same undercooling degrees. The effective distribution coefficient in the first crystallization stage was between 0.14–0.21 and 0.19–0.35 at the crystal growth rate between 2 × 10^–7^ and 6 × 10^–7^ m/s in binary solvent and single solvent systems, respectively. Furthermore, the crystal growth rate increased by a factor of 1.5 to 2 at lower DEG content in the second crystallization stage, indicating that the effective depletion and transport of DEG from the crystal surface to the bulk melt can enhance the crystal growth rate and purity. Therefore, the lower viscosity of the mother liquor in the binary solvent system favors crystal growth and higher purity. The formation of two liquid phases was observed in both single and binary solvent systems during crystallization. However, the purity of the final product up to 80% crystallization yield showed a good separation efficiency despite the formation of two liquid phases.

## Supporting Information Available

Additional experimental details including a schematic diagram of the cooling profile and figures illustrating the formation of the second liquid phase in the cold finger setup, and a validation method of HPLC analysis (PDF)
