# Supplementary material for: Effects of Assisting Solvents on Purification Efficiency in the Layer Melt Crystallization of Glycerol
Source: Cryst Growth Des. 2024 Feb 21;24(5):1952–8. doi: 10.1021/acs.cgd.3c01207 (PMC10921978; doi:10.1021/acs.cgd.3c01207)
Supplement: Supplementary file 1 — cg3c01207_si_001.pdf [file cg3c01207_si_001.pdf]

Supporting Information

Effects of Assisting Solvents on Purification Efficiency in the Layer Melt Crystallization of Glycerol

Mitra Ila[1], Kim Miikki[2], Marjatta Louhi-Kultanen[*1]

[1]Department of Chemical and Metallurgical Engineering, School of Chemical Engineering, Aalto University, Finland

[2]School of Chemical Engineering, Aalto University, Finland

[*]Corresponding author: marjatta.louhi-kultanen@aalto.fi

The following schematic diagram shows the cooling profile in the cold finger setup over a specified time period. During this process, the growth rate was monitored using the image analysis method.

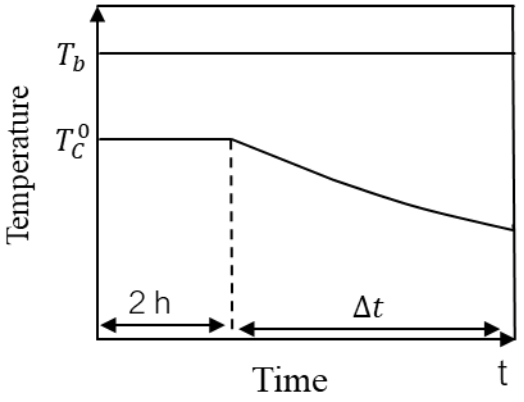

Figure S1. Schematic diagram of cooling profile in the cold finger setup over time. T_b and T_c are the bulk melt and cold finger temperature, respectively.

A thin layer of the second liquid phase was observed above the main body of the bulk melt during crystallization from the solvent-aided mixtures. The exact time required for the formation of a stable layer of the second phase is dependent on the temperature and concentration profiles within the melt.

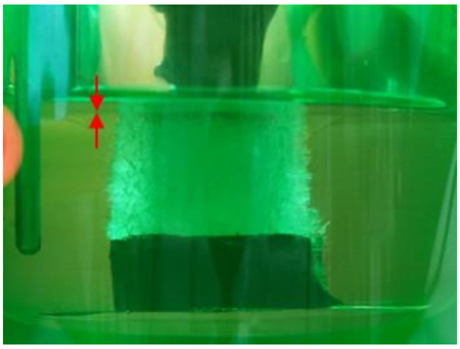

Figure S2. First crystallization stage in the single solvent system at initial undercooling of 15 degrees.

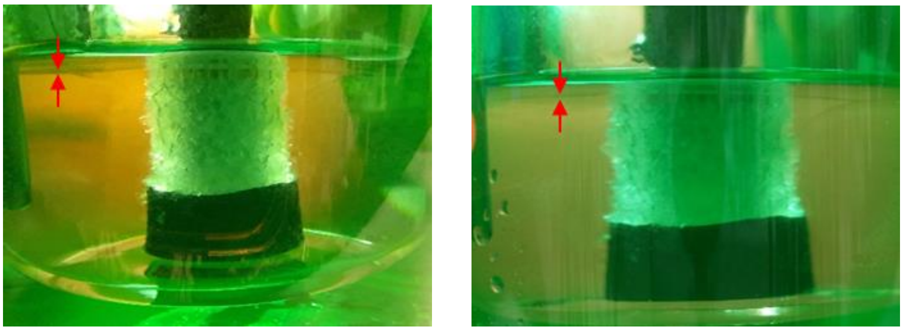

Figure S3. Second crystallization stage in the single solvent system at initial undercooling of 5 degrees.

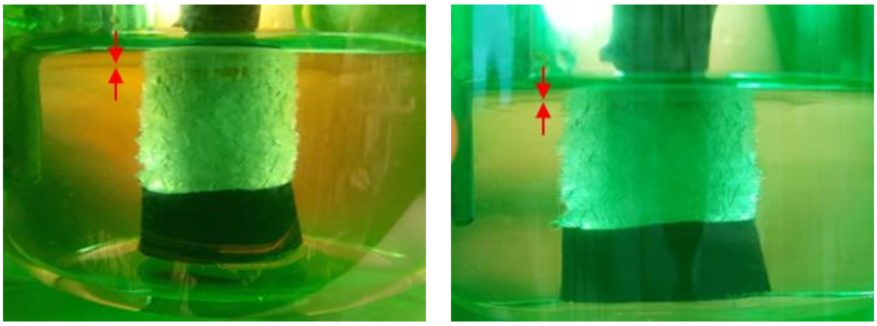

Figure S4. Second crystallization stage in the single solvent system at initial undercooling of 10 degrees.

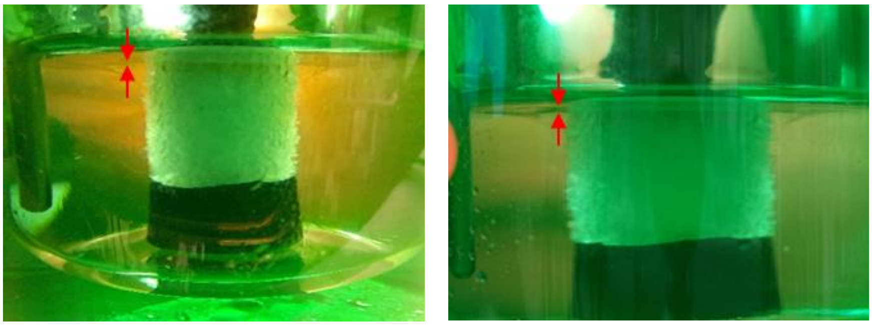

Figure S5. Second stage crystallization in the single solvent system at initial undercooling of 15 degrees.

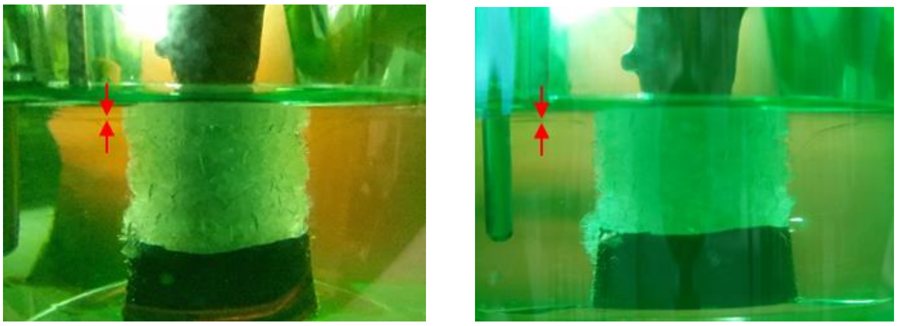

Figure S6. Second crystallization stage in the binary solvent system at initial undercooling of 5 degrees.

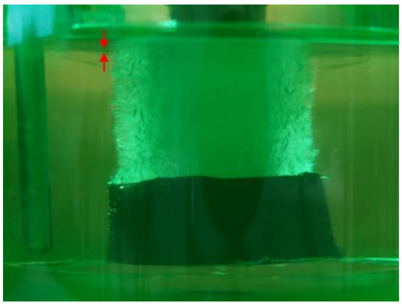

Figure S7. First crystallization stage in the binary solvent system at initial undercooling of 15 degrees.

Validation of HPLC method

To develop a calibration method in terms of the linearity of the calibration curve in the concentration range and repeatability, the calibration samples containing the binary mixture of glycerol and DEG with a concentration range between 0.96 wt.% and 0.998 wt.% were prepared and dissolved in water with a binary mixture:water ratio of 30 wt.%:70 wt.%. The results showed that the detector response of each individual component (peak area) was non-linear in the concentration range examined (Figure S8 (a)). However, Figure S8 (b) shows a linear relationship between the mass fraction of glycerol, w_{GL}, in standard samples and the relative HPLC peak area calculated using the following equation:

$$w_{GL-HPLC} = \frac{Peak\ area\ of\ glycrol}{Peak\ area\ of\ glycerol + Peak\ area\ of\ DEG}$$

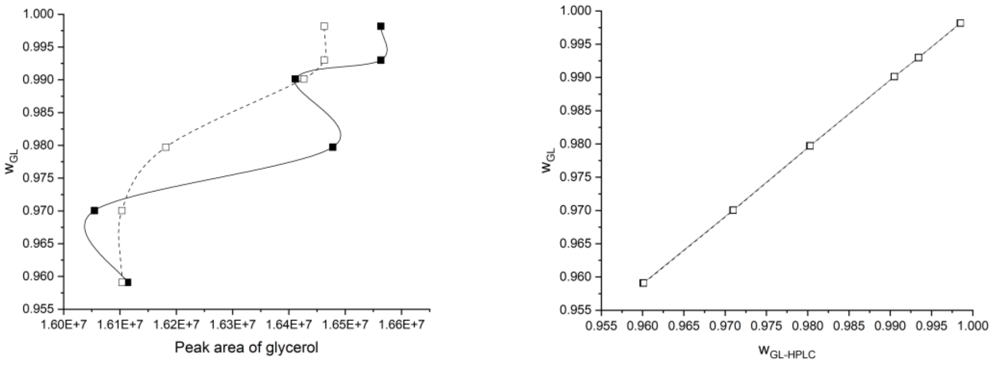

a) b)

Figure S8. Calibration curve for glycerol-DEG binary mixture. a) composition of the calibration samples against peak area of glycerol b) composition of the calibration samples against relative peak area of HPLC. Results from the first and second HPLC trials are illustrated by the solid and dashed lines, respectively.

The results obtained from the relative peak area showed a good consistency over multiple runs. To ensure the accuracy of the binary calibration curve to determine the purity of glycerol in the presence of traces of 1-butanol and acetone, samples with 10 mol.% of 1-butanol and 10 mol.% of acetone were analyzed using the same calibration method. The measured concentration of glycerol on a solvent-free basis deviated less than 0.05 wt.% from the actual concentration of glycerol in the samples. This deviation increased with increasing DEG concentration. Therefore, this method was applied for mixtures with less than 4 wt.% (solvent-free basis) of DEG.
